# Supplementary material for: Chromatin profiling in human neurons reveals aberrant roles for histone acetylation and BET family proteins in schizophrenia
Source: Nat Commun. 2022 Apr 22;13:2195. doi: 10.1038/s41467-022-29922-0 (PMC9033776; doi:10.1038/s41467-022-29922-0)
Supplement: Supplementary file 1 — Supplementary Information [file 41467_2022_29922_MOESM1_ESM.pdf]

Supplementary Information File

Chromatin profiling in human neurons reveals aberrant roles for histone acetylation and BET family proteins in schizophrenia

Lorna A. Farrelly, Shuangping Zheng, Nadine Schrode, Aaron Topol, Natarajan V. Bhanu, Ryan M. Bastle, Aarthi Ramakrishnan, Jennifer C Chan, Bulent Cetin, Erin Flaherty, Li Shen, Kelly Gleason, Carol A. Tamminga, Benjamin A. Garcia, Haitao Li, Kristen J. Brennand, Ian Maze

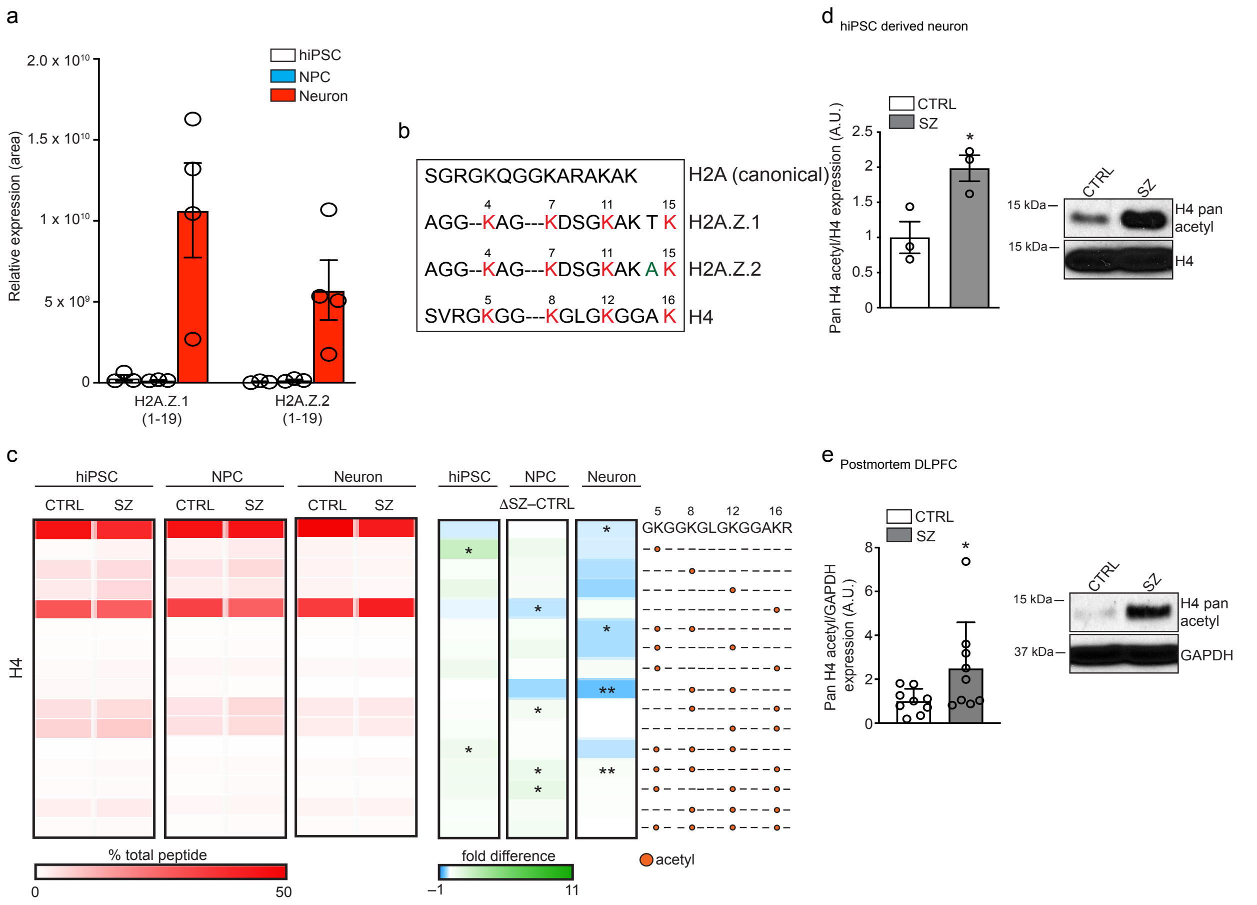

Supplementary Figure 1: H4 hyper-acetylation in SZ neurons.

(a) Relative expression of histone variant proteins H2AZ.1 and H2AZ.2 across control (non-disease) hiPSCs ($n = 3$), NPCs ($n = 3$) and neurons ($n = 4$). **(b)** Amino acid sequence comparisons of the N-terminal tails of histone H2A.Z.1/2 and histone H4. **(c)** Heatmap depicting LC-MS/MS data for relative enrichment values of (un)modified and acetylated histone H4 in hiPSCs (n = 3/group), NPCs (n = 3/group) and neurons from SZ cases and matched controls (n = 4/group). Absolute values (% total peptide) for each peptide are provided. Fold differences between SZ *vs.* controls are represented (biologically independent replicates/cell-type/condition). Heatmap data represented as means, *p≤0.05, **p≤0.01 (two-tail Student's t-tests performed within cell-type, SZ *vs.* CTRL; adjustments were not made for multiple comparisons). Please see **Supplementary**

Data 1 for LC-MS/MS source data. Increased patterns of H4 acetylation were confirmed via western blotting in **(d)** 4-week-old hiPSC neurons [*n* = 3 (SZ) *vs.* 3 (CTRL) biologically independent replicates], *p=0.0278 (two-tail Student's t-tests), and in **(e)** DLPFC from biologically independent postmortem SZ subjects *vs.* matched controls (*n* = 9 per group; two-tail Student's t-tests), *p=0.0565. A.U. = Arbitrary Units (normalized to CTRL samples). Data are presented as averages ± SEM. Source data are provided in Source Data files.

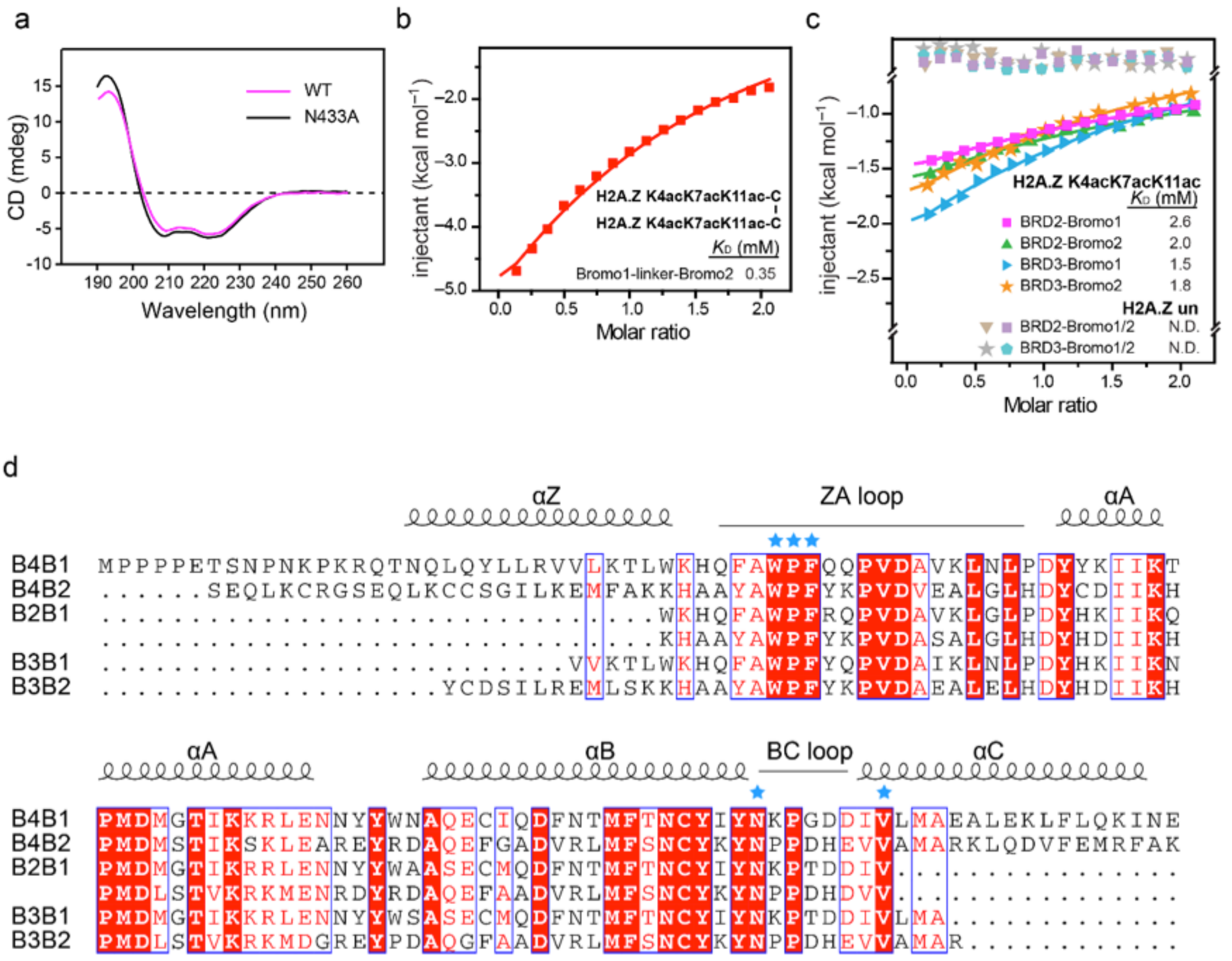

Supplementary Figure 2: BET family protein interactions with H2A.Zac

(a) CD spectra of $BRD4_{Bromo2}$WT (magenta) and $BRD4_{Bromo2}$N433A (black). The CD results indicate that $BRD4_{Bromo2}$N433A adopts the same conformation as $BRD4_{Bromo2}$WT. **(b)** ITC fitting curve of Bromo1-linker$(GGS)_5$-Bromo2 of BRD4 titrated with cross-linked H2A.ZK4ac7ac11ac peptides via the C-terminal cysteine. Compared with Bromo2 and Bromo1 of BRD4, the linked Bromo1-linker-Bromo2 of BRD4 increases binding of BRD4 towards polyacetylated H2A.Z by 1.7-and 6.49-fold, respectively. **(c)** ITC fitting curves of BRD_{Bromos} titrated with H2A.ZK4ac7ac11ac peptide *vs.* unmodified H2A.Z peptides. As expected, $BRD2_{Bromo1/2}$ and $BRD3_{Brono1/2}$ can also bind to polyacetylated H2A.Z. This is consistent with

sequence alignment analysis **(d)**. Conserved key residues responsible for polyacetylated H2A.Z recognition are labeled with blue stars.

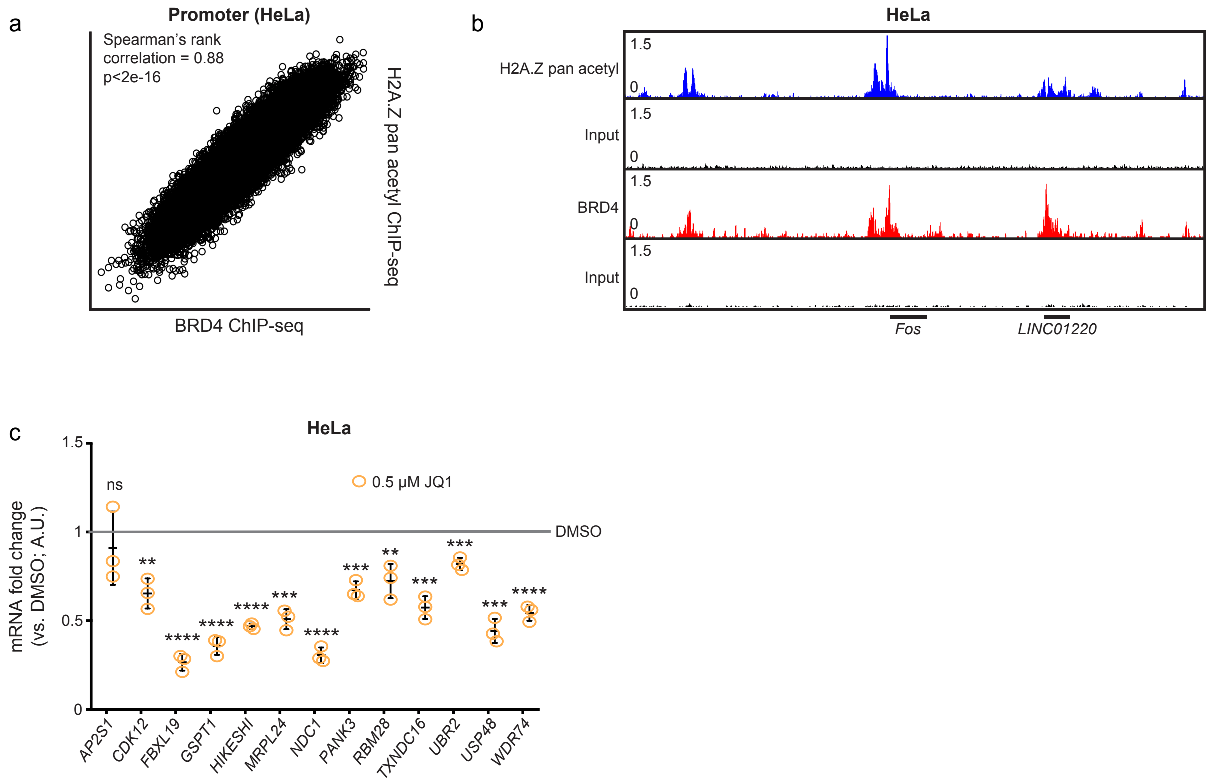

Supplementary Figure 3: H2A.Zac enrichment genome-wide strongly correlates with BRD4 occupancy in human cells

(**a**) Corrgram plot depicting correlations between H2A.Zac (*n*=3) and BRD4 (extracted from GSE151038) enrichments genome-wide at promoters in HeLa cells. Both Spearman's rank correlation coefficients and respective p-values are provided. (**b**) Example Integrative Genomics Viewer (IGV) tracks for H2A.Zac and BRD4 enrichment at *Fos* and *LINC01220* loci, demonstrating that BRD4 and H2A.Zac genomic enrichment profiles strongly overlap. A Respective input track is provided. (**c**) qPCRs (*n*=3/group) for genes co-enriching for H2A.Zac and BRD4 in HeLa cells –/+ JQ1, validating that these co-enriched loci are sensitive to BET family protein inhibition. Data were normalized to DMSO controls. **$p \leq 0.01$, ***$p \leq 0.001$,

****$p \leq 0.0001$ (two-tail Student's t-tests). A.U. = Arbitrary Units (normalized to DMSO samples).

Data are presented as averages ± SEM. Source data are provided in Source Data files.

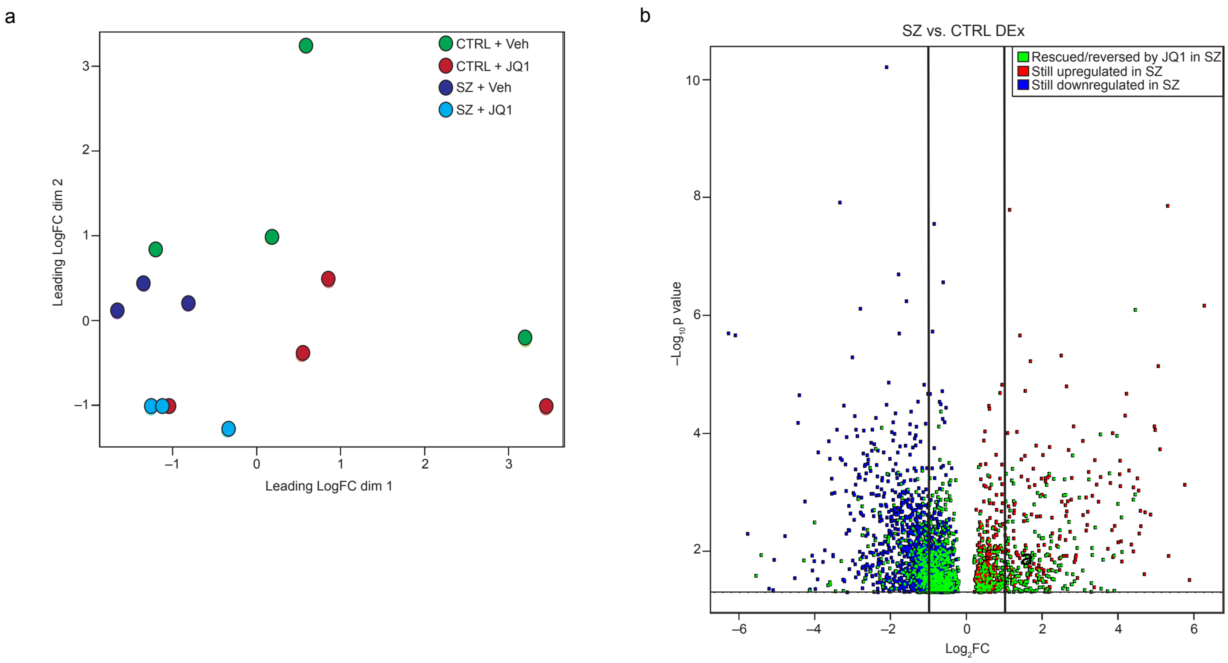

Supplementary Figure 4: RNA-seq of SZ (SZ), control (CTRL) and JQ1-treated hiPSC-neurons.

(**a**) Multidimensional scaling plot of gene expression log2FCs in all samples. (**b**) Volcano plot of differential expression in SZ hiPSC-neurons compared to controls, with significantly differentially expressed genes in SZ highlighted in blue and red, and genes whose differential expression was reversed by JQ1 treatment in green.

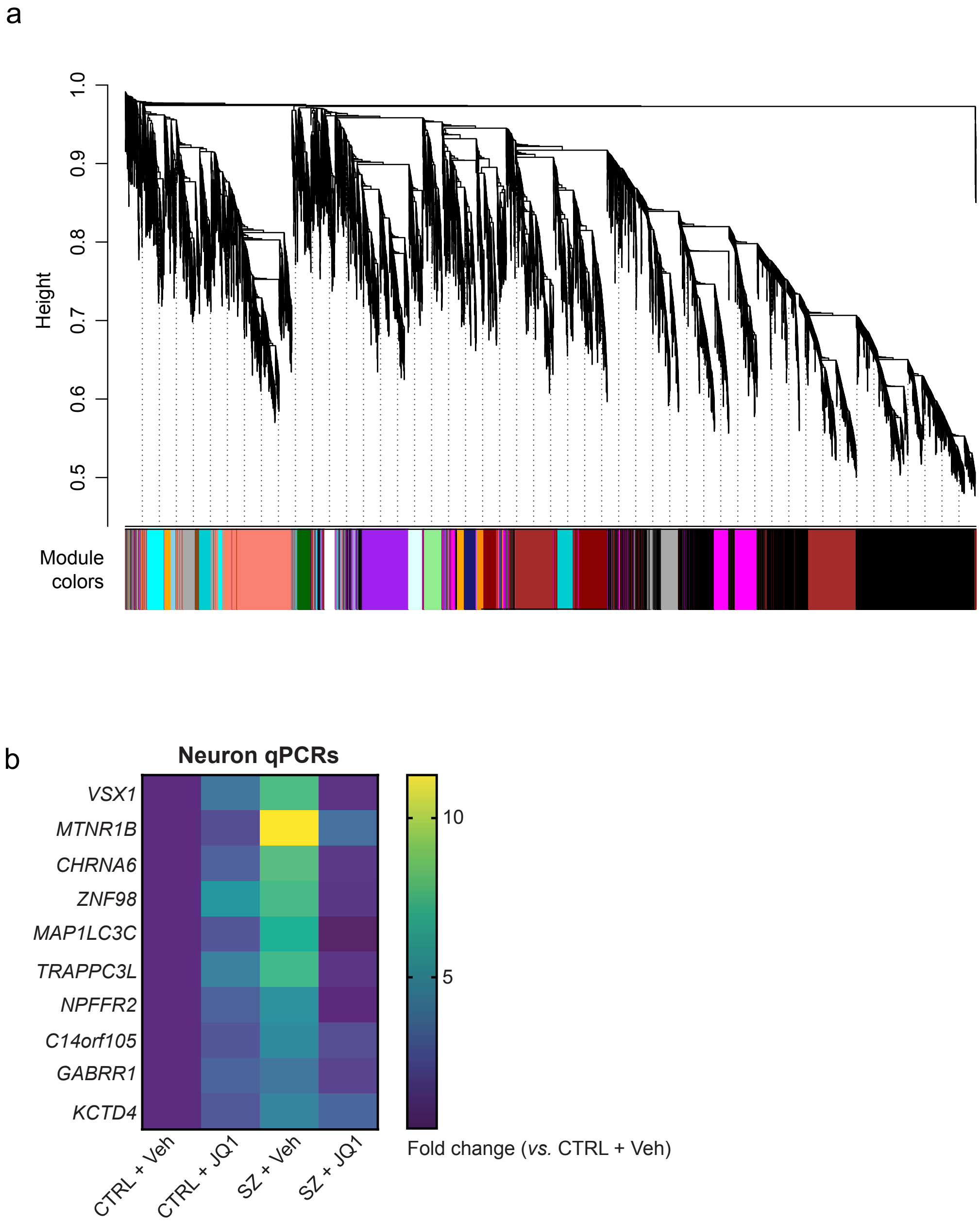

Supplementary Figure 5: WGCNA and synergistic gene expression effects by JQ1

(**a**) Hierarchical clustering of genes through topological overlap-based dissimilarity (top) and assigned module colors (bottom). (**b**) qPCRs of ten candidate SZ regulated genes (from RNA-seq) in hiPSC derived SZ *vs.* control neurons –/+ JQ1. *n* = 3 (SZ) - 4 (CTRL) biologically independent

replicates/condition. Heatmap data represented as means (normalized to CTRL + Veh). All genes were normalized to the housekeeping gene *18S*. Two-way ANOVA Interaction (SZ and JQ1) p-values: *VSX1* (0.0625), *MTNR1B* (0.0710), *CHRNA6* (0.0075), *ZNF98* (0.0641), *MAP1LC3C* (0.0422), *TRAPPC3L* (0.0758), *NPFFR2* (0.0643), *C14orf105* (0.0355), *GABRR1* (0.1149), *KCTD4* (0.0952). Source data are provided in Source Data files.

	Healthy controls (n=9)	Patients (n=9)
**Age**	58.7	47.7
**Sex (male, %)**	8 (88.8%)	6 (66.6%)
**PMI**	19.27	15.3
**RIN**	9.01	8.6
**BMI**	29.5	31.0
Race		
**Caucasian**	8	5
**African American**	0	4
**Other**	1	0

Supplementary Table 1: Demographic Parameters for Postmortem Healthy Controls and Patients

Data Collection	
Space group	$P2_12_12$
Unit Cell	
a, b, c (Å)<br>α, β, γ (°)	59.2, 74.0, 32.4<br>90, 90, 90
Resolution (Å)	50-1.5 (1.53-1.50)
No. of unique reflections	22,898 (1132)
R_{sym} (%)	4.1 (8.2)
R_{pim} /CC1/2 (%)	1.6 (3.4)/99.6(99.7)
$I/\sigma (I)$	63.7 (37.7)
Completeness (%)	98.0 (98.3)
Redundancy	7.5 (7.8)
**Refinement (F>0)**	
Resolution (Å)	24.3-1.5
No. of unique reflections	22,859
R_{work}/R_{free} (%)	15.6/17.7
No. of non-H atoms	
Protein	959
Peptide	51
Water	177
Average B-factors ($Å^2$)	
Protein	16.6
Peptide	44.4
Water	32.2
RMSD bonds (Å)	0.011
RMSD angle (°)	1.253
Ramachandran plot (%)	
Most favored	96.0
Additional allowed	4.0

Supplementary Table 2: Crystallography Data Collection and Refinement Statistics

Gene	Forward Primer	Reverse Primer
**VSX1**	GGTCTGGACAGCAGAGGAAG	GGGAGCTCAGTTTTCACAGC
**MTNR1B**	CAAAGCCCTCTTGTCAAAGC	AGCCAGGTCCTATGTGGATG
**CHRNA6**	GCTTCTTCCCTCAGTGTTGC	CCTTGCAGCTGGAGGTAGTC
**ZNF98**	AGCCCAAAAGCTTGAGAACA	GGTGCATGGCACAATTACAG
**MAP1LC3C**	GCTGAGATCGTGCCACTACA	GCTAGGTGTCCTCCAATCCA
**TRAPPC3L**	AGTTATGGACCTGCCCACTG	CTGGGAGCAGCATGTGACTA
**NPFFR2**	GAGTCAGTGGGCTGAGGAAG	GCAAGGAGAGCCAGTTTGAC
**C14orf105**	TCTGAAATGGGGAATCTTCG	ATTTGTGCCATCCTCTGGAC
**GABRR1**	GGCTCGAGAACTACGTGAGG	TGGAGGCTTTGCTCATTTCT
**KCTD4**	CTTTCAGCTCAAGGGACTGG	CTCCTCTGGAAATCCATCCA
**18S**	AAACGGCTACCACATCCAAG	CCTCCAATGGATCCTCGTTA

Supplementary Table 3: Human qPCR Primers
