# Supplementary material for: Chromatin profiling in human neurons reveals aberrant roles for histone acetylation and BET family proteins in schizophrenia
Source: Nat Commun. 2022 Apr 22;13:2195. doi: 10.1038/s41467-022-29922-0 (PMC9033776; doi:10.1038/s41467-022-29922-0)
Supplement: Supplementary file 3 — Description of additional Supplementary File [file 41467_2022_29922_MOESM3_ESM.pdf]

Description of additional Supplementary data files

Supplementary Data 1: LC-MS/MS quantifications of histone peptide/PTM ratios for hiPSCs, NPCs and neurons (4-week) comparing SZ *vs.* controls.
