# Supplementary material for: Chromatin profiling in human neurons reveals aberrant roles for histone acetylation and BET family proteins in schizophrenia
Source: Nat Commun. 2022 Apr 22;13:2195. doi: 10.1038/s41467-022-29922-0 (PMC9033776; doi:10.1038/s41467-022-29922-0)
Supplement: Supplementary file 5 — Reporting Summary [file 41467_2022_29922_MOESM5_ESM.pdf]

nature research

Corresponding author(s): Ian Maze, Kristen J. Brennand, Haitao Li

Last updated by author(s): January 25, 2022

Reporting Summary

Nature Research wishes to improve the reproducibility of the work that we publish. This form provides structure for consistency and transparency in reporting. For further information on Nature Research policies, see Authors & Referees and the Editorial Policy Checklist .

Statistics

For all statistical analyses, confirm that the following items are present in the figure legend, table legend, main text, or Methods section.

n/a	Confirmed	
☐	☒	The exact sample size (n) for each experimental group/condition, given as a discrete number and unit of measurement
☐	☒	A statement on whether measurements were taken from distinct samples or whether the same sample was measured repeatedly
☐	☒	The statistical test(s) used AND whether they are one- or two-sided<br>*Only common tests should be described solely by name; describe more complex techniques in the Methods section.*
☒	☐	A description of all covariates tested
☒	☐	A description of any assumptions or corrections, such as tests of normality and adjustment for multiple comparisons
☐	☒	A full description of the statistical parameters including central tendency (e.g. means) or other basic estimates (e.g. regression coefficient) AND variation (e.g. standard deviation) or associated estimates of uncertainty (e.g. confidence intervals)
☐	☒	For null hypothesis testing, the test statistic (e.g. F, t, r) with confidence intervals, effect sizes, degrees of freedom and P value noted<br>*Give P values as exact values whenever suitable.*
☒	☐	For Bayesian analysis, information on the choice of priors and Markov chain Monte Carlo settings
☒	☐	For hierarchical and complex designs, identification of the appropriate level for tests and full reporting of outcomes
☐	☒	Estimates of effect sizes (e.g. Cohen's d, Pearson's r), indicating how they were calculated

Our web collection on statistics for biologists contains articles on many of the points above.

Software and code

Policy information about availability of computer code

Data collection

No custom algorithms were used.

Data analysis

For most statistical comparisons, Prism 9 [Version 9.3.1 (350)) for macOS Mojave (Version 10.14.6) was used.

Histone PTM LC-MS/MS
Data were analyzed using the in-house (Garcia Lab) software, EpiProfile, wherein peptide relative ratios were calculated using the total area under the extracted ion chromatograms of all peptides with the same amino acid sequence (including all of their modified forms) as 100%.

RNA sequencing
All RNA-Seq analyses were performed in R (www.R-project.org). All heat maps and their hierarchical clustering were computed using the pheatmap package. If the number of values to be clustered exceeded 1000, rows were pre-aggregated using k-means clustering.

Data preprocessing
Genes with over 15 counts in at least 2 samples were retained for further analysis. Normalization factors for library sizes were calculated using the trimmed mean of M-values (TMM) method in the calcNormFactors function (edgeR package). For the purpose of linear modeling, 'JQ treatment' and 'Diagnosis' were combined into one group variable.

Differential gene expression analysis
The limma voom function was used to compute weights for heteroscedasticity adjustment by estimating the mean-variance trend for log2 counts. Linear models were fit to the expression values of each gene using the lmFit function: Gene expression ~ group. The coefficients, standard deviations and correlation matrix were then recalculated, using contrasts.fit in terms of the comparisons of interest. Empirical Bayes moderation was applied using the eBayes function to obtain more precise estimates of gene-wise variability. P-values were adjusted for multiple hypotheses testing using false discovery rate (FDR) estimation, and differentially expressed genes were

determined as those with an estimated FDR ≤ 5%, unless stated otherwise.

Concordance analysis
Concordance between differential expression in the tested comparisons and one external dataset (post-mortem (CMC, common mind consortium) was evaluated through Spearman correlation of t-statistics.

Analysis of synergistic effects
The expected additive effect was modeled through addition of the individual comparisons: (JQ vs ctrl) + (SZ vs ctrl). The synergistic effect was modeled by subtraction of the additive effect from the combinatorial comparison: (SZ+JQ vs ctrl) - (JQ vs ctrl) - (SZ vs ctrl). Fitting of this model for differential expression gives genes that show a difference in the differential expression computed for an additive effect model and that computed for the combinatorial experiment. However, interpretation of the resulting DEGs depends on several factors, such as the direction of fold change in all three models. To identify genes of interest, namely those whose magnitude of change is larger in the combinatorial perturbation vs. the additive model, we categorized all genes by the direction of their change in both models and their log2FC in the synergistic model. First, log2FC standard errors (SE) were calculated for all samples. Genes were then grouped into 'positive synergy' if their FC was larger than SE and 'negative synergy' if smaller than -SE. If the corresponding additive model log2FC showed the same or no direction, the gene was classified as "more" differentially expressed in the combinatorial experiment than predicted. 1,115 genes were computed to be in this category (673 more down, 442 more up).

Enrichment analysis
Gene set enrichment analysis (GSEA) was performed on a curated subset of the MAGMA collection using the limma package camera function, which tests if genes are ranked highly in comparison to other genes in terms of differential expression, while accounting for inter-gene correlation. Due to the small sample size in this study and moderate fold changes overall, changes in gene expression may be small and distributed across many genes. However, similar to previous studies, more powerful enrichment analyses in the limma package were used. These evaluate enrichment based on genes that are not necessarily genome-wide significant, and identify sets of genes for which the distribution of t-statistics differs from expectation.

Over-representation analysis
Over-representation analysis (ORA) was performed when subsets of DEGs were of interest, such as the synergistic 'more up' and 'more down' genes. The genes of interests were ranked by –log10 (p-value) and enrichment was performed against a background of all expressed genes using the WebGestaltR package.

Weighted gene co-expression network analysis (WGCNA)
Unsigned co-expression networks were generated using the WGCNA R package from normalized and residualized expression data (17,792 genes) of all samples. An unsigned adjacency matrix was constructed using a soft thresholding power of 7 to maximize scale-free topology model fitting. The adjacency matrix was then transformed into a topological overlap matrix (TOM) to reduce noise, which in turn was transformed into a dissimilarity matrix by computing 1-TOM. Hierarchical clustering was performed on the TOM-based dissimilarity matrix. Finally, modules were identified using dynamic tree cut as a function of the hierarchical gene clustering and dissimilarity matrix. To merge modules with high similarity, module eigengenes (ME) were calculated, clustered as above and visually inspected for the minimum merging height cut-off, which was chosen at 0.17. The resulting 18 co-expression modules were then assigned a color and unconnected genes grouped into a "grey" module.

ChIP-sequencing
Raw sequencing reads from HeLa cell lines were mapped to hg38 using HISAT2. Uniquely mapped reads were retained. Alignments were filtered using SAMtools to remove duplicate reads, and peak-calling–normalized to respective inputs–was performed using MACSv2.1.1 with default settings and filtered for $p < 0.05$ and fold change > 1.2. Peaks called were annotated using region analysis software and saved in tab delimited text file.

Corrgram -

BRD4 DMSO samples were obtained from GSE151038. TSS sites for all genes in the genome were found using Ensembl BioMart. Promoter regions were defined as +/- 1kb from the TSS sites. Counts of reads mapping to the promoter regions for H2AZac and BRD4 samples were obtained using featureCounts. Raw counts were converted to RPKM and corrgram R package was utilized to compute spearman correlation between the coverage of promoter regions of BRD4 and H2AZac datasets.

The NGS-Data-Charmer repository, which hosts an automated NGS data analysis pipeline, was used in ChIP-seq analyses (https://github.com/shenlab-sinai/NGS-Data-Charmer).

For manuscripts utilizing custom algorithms or software that are central to the research but not yet described in published literature, software must be made available to editors/reviewers. We strongly encourage code deposition in a community repository (e.g. GitHub). See the Nature Research guidelines for submitting code & software for further information.

Data

Policy information about availability of data

All manuscripts must include a data availability statement. This statement should provide the following information, where applicable:

- Accession codes, unique identifiers, or web links for publicly available datasets
- A list of figures that have associated raw data
- A description of any restrictions on data availability

The RNA-seq data generated in this study have been deposited in the National Center for Biotechnology Information Gene Expression Omnibus (GEO) database under accession number GSE144639 (https://www.ncbi.nlm.nih.gov/geo/query/acc.cgi?acc=GSE144639). The atomic coordinates and structure factors have been deposited in the Protein Data Bank (PDB) under PDB ID code 6KO2 (https://www.rcsb.org/structure/6KO2). The histone modification mass spectrometry proteomics

data have been deposited to the ProteomeXchange Consortium via the PRIDE partner repository with the dataset identified PXD031767 (http://proteomecentral.proteomexchange.org/cgi/GetDataset?ID=PXD031767). We declare that the data supporting findings for this study are available within the article and Supplementary Information, and source data are provided with this paper (Source Data files 1 and 2). Related data are available from the corresponding author upon reasonable request. No restrictions on data availability apply.

Field-specific reporting

Please select the one below that is the best fit for your research. If you are not sure, read the appropriate sections before making your selection.

[x] Life sciences [ ] Behavioural & social sciences [ ] Ecological, evolutionary & environmental sciences

For a reference copy of the document with all sections, see nature.com/documents/nr-reporting-summary-flat.pdf

Life sciences study design

All studies must disclose on these points even when the disclosure is negative.

Sample size	Sample sizes were indicated in the legend of each Figure and Supplementary Figure. No statistical methods were used to predetermine sample sizes.
Data exclusions	No data were excluded.
Replication	All experiments were replicated at least twice. All attempts to replicate main findings were successful.
Randomization	All patient cells were maintained in the same environment and were randomly assigned to the experimental groups.
Blinding	Investigators were blinded to conditions in all experiments presented (e.g., sample IDs for blotting, LC-MS/MS, RNA-seq, etc.).

Reporting for specific materials, systems and methods

We require information from authors about some types of materials, experimental systems and methods used in many studies. Here, indicate whether each material, system or method listed is relevant to your study. If you are not sure if a list item applies to your research, read the appropriate section before selecting a response.

Materials & experimental systems

n/a	Involved in the study	
[ ]	[x]	Antibodies
[ ]	[x]	Eukaryotic cell lines
[x]	[ ]	Palaeontology
[x]	[ ]	Animals and other organisms
[ ]	[x]	Human research participants
[x]	[ ]	Clinical data

Methods

n/a	Involved in the study	
[ ]	[x]	ChIP-seq
[x]	[ ]	Flow cytometry
[x]	[ ]	MRI-based neuroimaging

Antibodies

Antibodies used

Western Blotting: Histone H2AZ (Active Motif; Cat#39113; Lot#06217001; 1:1,000), Histone H2AZ.pan Acetyl (Active Motif; Cat#39642; Lot#26409001; 1:500), Histone H2AZ.pan Acetyl (GeneTex; Cat#GTX60813; Lot#822002999; 7.5 ug/IP), Histone H4 (Abcam; Cat#Ab10158; Lot#GR3268080-1; 1:10,000), Histone H4K5K8K12acetyl (GeneTex; Cat#GTX60337; Lot No. 821900948: 1:500), GAPDH (Sigma Cat#G9545; Lot#015M4824V; 1:1,000).

Immunocytochemistry: The following primary antibodies and dilutions were used: goat anti-Nanog (R&D, AF1997), 1:200; mouse anti-Tra1-60 (Millipore, MAB4360), 1:100; mouse anti-human Nestin (Millipore, ABD69), goat anti-Sox2 (Santa Cruz, sc-17320), 1:200; rabbit anti-βIII-tubulin (Covance, PRB-435P), 1:200; mouse anti-MAP2AB (Sigma, M1406), 1:200; mouse anti-S100b (Sigma-Aldrich, S2532), 1:1000. Secondary antibodies were Alexa donkey anti-rabbit 488 (Jackson Immuno 711-545-152) and 568 (Life Technologies A10042), Alexa donkey anti-mouse 488 (Jackson Immuno 715-545-151) and 568 anti-mouse (Life Technologies A10037), and Alexa donkey anti-goat 488 (Jackson Immuno 705-545-147) and 568 (Jackson Immuno 705-605-147); all were used at 1:300.

Validation

All antibodies used in this study (all of which have been commercially validated) were validated in human cell lines or tissues via immunoblotting or IHC/IF prior to experimentation.

Eukaryotic cell lines

Policy information about cell lines

Cell line source(s)	Fibroblasts from four cases and four controls were obtained from the Coriell Cell Repository (Camden, NJ, USA) or American Type Culture Collection (Manassas, VA, USA) and reprogrammed as described previously using tetracycline-inducible lentiviruses expressing OCT4, SOX2, KLF4, cMYC and LIN28, driven by the reverse tetracycline transactivator (rtTA).<br><br>HeLa cell lines were obtained from the American Type Culture Collection (ATCC).
Authentication	hPSCs and derived NPCs/forebrain neurons, along with HeLa cells, were validated by immunocytochemical staining for cell-type specific markers and imaged for appropriate morphology.
Mycoplasma contamination	We can confirm that all cell lines tested negative for mycoplasma contamination.
Commonly misidentified lines<br>(See ICLAC register)	No commonly misidentified cell lines were used.

Human research participants

Policy information about studies involving human research participants

Population characteristics	Population characteristics for hiPSC lines can be found in the Methods, and demographic information for postmortem human samples can be found in Supplementary Table 1
Recruitment	hiPSC, NPC and Neuron culture<br><br>Fibroblasts from four cases and four controls were obtained from the Coriell Cell Repository (Camden, NJ, USA) or American Type Culture Collection (Manassas, VA, USA) – all de-identified, so IRB approval at the ISMMS was not necessary.<br><br>Human postmortem tissues<br><br>Human brain tissues from the UT Neuropsychiatry Research Program (Dallas Brain Collection-DBC) were collected from the Southwestern Institute of Forensic Sciences at Dallas, UT Southwestern Transplant Services Center, and UT Southwestern Willed Body Program; after consent from donor subjects' next of kin was received, along with permission to access medical records and to hold a direct telephone interview with a primary caregiver. All clinical and medical information obtained for each donor is reviewed by a three research psychiatrists, using DSM-V criteria for diagnoses. Blood toxicology screens for drugs of abuse, alcohol and prescription drugs, including psychotropics, are conducted for each donor subject from the Southwestern Institute of Forensic Sciences at Dallas.
Ethics oversight	Collection of postmortem human brain tissues is approved by the University of Texas Southwestern Medical Center Institutional Review Board [STU 102010-053].

Note that full information on the approval of the study protocol must also be provided in the manuscript.

ChIP-seq

Data deposition

- [x] Confirm that both raw and final processed data have been deposited in a public database such as GEO.
- [x] Confirm that you have deposited or provided access to graph files (e.g. BED files) for the called peaks.

Data access links<br>*May remain private before publication.*	https://www.ncbi.nlm.nih.gov/geo/query/acc.cgi?acc=GSE144639; Reviewer token - ytevmqsevdgnbez
Files in database submission	h2azac_peaks.bed.gz<br><br>annotated_h2azac_peaks.txt.gz<br><br>3_S3_L001_R1_001.fastq.gz<br><br>4_S4_L001_R1_001.fastq.gz<br><br>7_S7_L001_R1_001.fastq.gz<br><br>8_S8_L001_R1_001.fastq.gz<br><br>2__S1_L001_R1_001.fastq.gz<br><br>6__S4_L001_R1_001.fastq.gz

	3_S3_L001_R2_001.fastq.gz<br><br>4_S4_L001_R2_001.fastq.gz<br><br>7_S7_L001_R2_001.fastq.gz<br><br>8_S8_L001_R2_001.fastq.gz
Genome browser session (e.g. UCSC)	https://ramaka02.u.hpc.mssm.edu/H2AZac_Tracks/

Methodology

Replicates	3 replicates for H2A.Zac samples; 3 respective inputs
Sequencing depth	35 million reads; 23 million uniquely mapped reads; length of reads: 51; single and paired-end
Antibodies	Histone H2AZ.pan Acetyl (GeneTex; Cat#GTX60813; Lot#822002999)
Peak calling parameters	HISAT2 v2.2.1 aligner with default parameters utilized for read mapping against Ensembl v90 hg38 genome. MACSv2.1.0 with default parameters used to call peaks. ChIP: H2A.Zac IP samples; Control: H2A.Zac Input samples
Data quality	30282 peaks called at q<0.05 and fold change > 5
Software	NGS-Data-Charmer: https://github.com/shenlab-sinai/NGS-Data-Charmer
