# Supplementary material for: Chromatin profiling in human neurons reveals aberrant roles for histone acetylation and BET family proteins in schizophrenia
Source: Nat Commun. 2022 Apr 22;13:2195. doi: 10.1038/s41467-022-29922-0 (PMC9033776; doi:10.1038/s41467-022-29922-0)
Supplement: Supplementary file 6 — Source data [file 41467_2022_29922_MOESM6_ESM.zip › NCOMMS-20-04109B_Source data/242037_2_related_ms_6313864_r7nvvd.pdf]

hiPSCs, NPCs & Neurons (4-week)
LC-MS/MS Quantifications of H2A.Z.1/2
Figure. 1b
Relevant Quantifications (K4/K7/K11) Provided

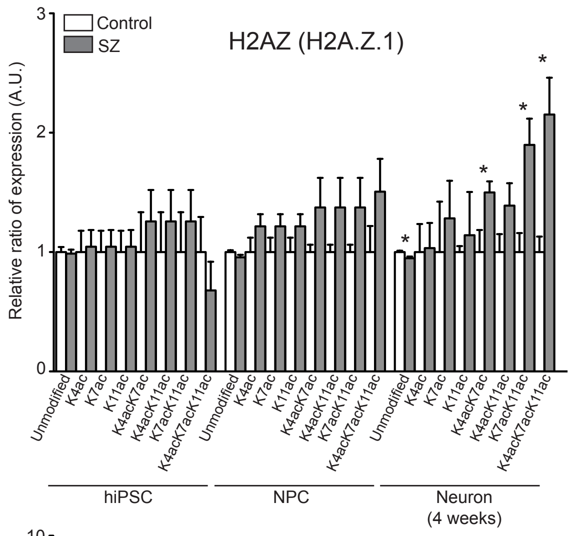

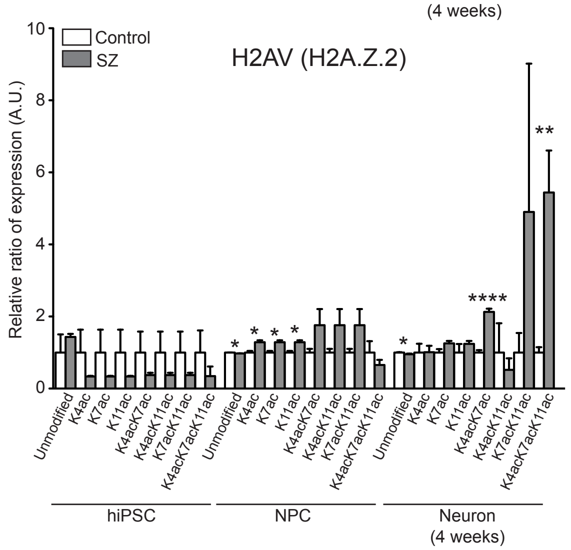

hiPSC Neurons (4-week)
C=CTRL, S=SZ
Figure. 1c and Supplemental Figure 1d

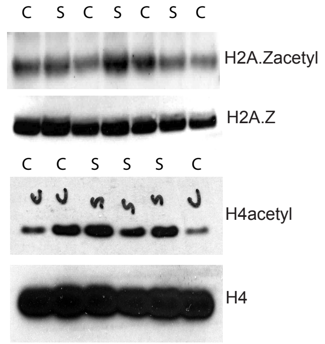

Postmortem DLPFC
1=CTRL, 2=SZ
Figure 1d and Supplemental Figure 1e

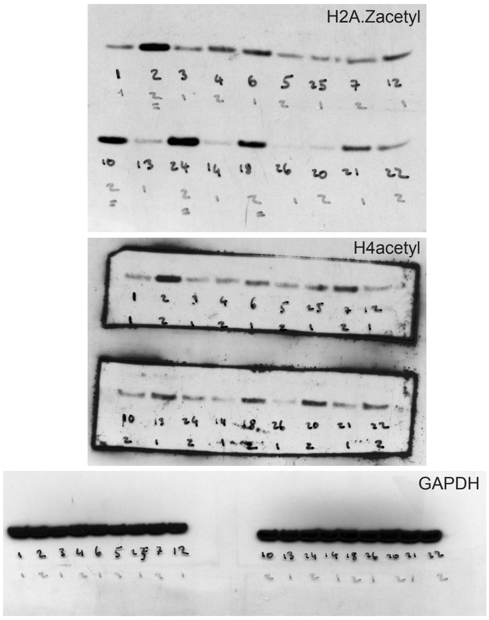

Source Data 1: Box plot representations of H2A.Z LC-MS/MS data and uncropped western blots
